# Employment and Its Determinants for Spinal Cord Injury Population in Romania

**DOI:** 10.3390/neurosci6010010

**Published:** 2025-02-01

**Authors:** Camelia Florentina Lascu, Daiana Popa, Camelia Liana Buhaș, Florica Voiţă-Mekereş, Florina Ligia Popa, Matei Teodorescu, Georgiana Albina Căiță, Gheorghe Szilagyi, Andrada Florina Schwarz-Madar, Mirela Elena Bodea, Călin Tudor Hozan

**Affiliations:** 1 Doctoral School of Biomedical Sciences, Faculty of Medicine and Pharmacy, University of Oradea, 1 Universitatii Street, 410087 Oradea, Romania; camelu5h_13@yahoo.it (C.F.L.); cameliabuhas@uoradea.ro (C.L.B.); georgiana.caita@didactic.uoradea.ro (G.A.C.); schwarz_andrada@yahoo.com (A.F.S.-M.); mirelabodea16@gmail.com (M.E.B.); 2 Băile Felix Medical Rehabilitation Hospital, 417500 Băile Felix, Romania; 3 Morphological Disciplines Department, Faculty of Medicine and Pharmacy, University of Oradea, 1 Universitatii Street, 410087 Oradea, Romania; 4 County Clinical Emergency Hospital of Oradea, 410169 Oradea, Romania; gszilagyi@uoradea.ro; 5 Department of Physical Medicine and Rehabilitation, Faculty of Medicine, Lucian Blaga University of Sibiu, 550169 Sibiu, Romania; florina-ligia.popa@ulbsibiu.ro; 6 Rehabilitation Department, Elias Emergency Hospital, 011461 Bucharest, Romania; matei.teodorescu@umfcd.ro; 7 Surgical Disciplines Department, Faculty of Medicine and Pharmacy, University of Oradea, 1 Universitatii Street, 410087 Oradea, Romania; hozancalin@yahoo.com

**Keywords:** spinal cord injury, Romanian national spinal cord injury survey, employment, determinants, rehabilitation, integration

## Abstract

This study investigates employment rates and their determinants among individuals with spinal cord injury (SCI) in Romania using data from the Romanian National Spinal Cord Injury Survey (RO-InSCI), part of the International Spinal Cord Injury (InSCI) Community Survey. The cross-sectional study included 215 adults with traumatic or non-traumatic SCI living in the community. Participants were recruited through rehabilitation hospitals and patient organizations. Employment status, demographic characteristics, and injury-related factors were assessed. The observed employment rate was 25.35%, with a 39.45% employment gap compared to the general population. Barriers to employment included health status, disability, inadequate transport, and insufficient access to infrastructure, particularly for those with traumatic SCI. Vocational rehabilitation participation was low (18.7%), despite a strong desire to return to work (82% for traumatic SCI, 61.1% for non-traumatic SCI). Before injury, participants were primarily employed in elementary occupations, with higher rates among those with paraplegia. The findings highlight the need for targeted interventions, inclusive labor market policies, and improved accessibility to vocational rehabilitation to support workforce reintegration and address the specific needs of individuals with SCI in Romania.

## 1. Introduction

According to WHO statistics, between 250,000 and 500,000 individuals suffer a spinal cord injury (SCI) each year [1]. SCI can be either traumatic, caused by mechanical shocks (falls, violence, car accidents, etc.), or non-traumatic, caused usually by infections or cancer [2]. Employment is a key indicator of successful rehabilitation and community integration for people with disabilities, including those with SCI. Many of these individuals leave their jobs due to disability and health-related challenges. Only a small proportion of people with health-related disabilities manage to retain or return to employment in OECD member countries [3]. Employed SCI individuals experience higher levels of life satisfaction, sense of purpose, mental stimulation, social contact, and overall well-being [4]. From a sociological perspective, being productive through employment also contributes to a country’s social well-being [5]. However, the worldwide average employment rate among people with SCI is approximately 35% to 37%, with only 12% returning to their pre-injury jobs [6,7,8].

The level of employment among people with SCI is positively influenced by a range of factors, including personal characteristics (younger age at the time of injury, higher educational level, and higher motivation), SCI-related characteristics (less severe neurological impairment), and employment-related factors (employer support and the possibility of continuing work within the same organization) [9]. The importance of employment for individuals with SCI was first raised in 1959 [10] and is consistently identified as a priority post-injury. The International Classification of Functioning, Disability, and Health (ICF) defines employment as “engaging in all aspects of work, as an occupation, trade, profession or other forms of employment, for payment or where payment is not provided, as an employee, full or part-time, or self-employed” [11].

Research indicates that a considerable number of unemployed individuals with SCI want to work and perceive themselves as capable of working [12]. However, only a limited proportion of SCI individuals, including those who were employed prior to injury, are actually employed post-injury [13]. To improve employment outcomes for SCI individuals, a thorough understanding of the factors influencing these outcomes is essential.

Currently, there are few studies on post-SCI employment in the literature. One such study, based on the InSCI Community Survey, included data from 9785 individuals with SCI across 22 countries, including Romania, and reported an observed global employment rate of 38% among SCI individuals. A significant variation was found across countries, with employment rates ranging from 10.3% to 61.4%. Disparities between SCI individuals’ employment rates and those of the general population were considerable, ranging from 14.8% to 54.8%. On average, employment rates were slightly higher for men than women, though substantial variations existed across countries [14]. The variation in employment rates suggests that factors such as job opportunities for people with disabilities, adapted work environments, employment policies, and cultural perspectives on disability and work likely affect employment outcomes. Commonly cited factors influencing employment rates among SCI individuals include education, job type, disability degree, age, time since injury, sex, marital status, social support, vocational counseling, SCI-related medical issues, employer role, environment, and professional interests [12]. This evidence underscores that employment outcomes are a complex interaction between injury-related and contextual (personal/environmental) factors [5]. The disparities between SCI individuals’ employment rates and those of the general population underscore the need for more inclusive labor market policies in many countries [14].

Although global employment data highlight important trends, understanding the situation within individual countries is essential for identifying specific barriers and solutions. Romania’s case, in particular, reveals challenges that further emphasize the importance of localized approaches to improving employment outcomes for individuals with disabilities.

In Romania, general data on people with disabilities are sporadic and inconsistent. A study conducted in 2009 indicated that the employment rate for people with disabilities was significantly lower than that of the general population, with only 12.7% of disabled individuals aged 18–55 employed, compared to 70% for the general population in the same age range. Despite a positive trend since the early 2000s, where employment rates for disabled individuals doubled between 2003 and 2009 (with an absolute increase of threefold in the number of employed individuals with disabilities), Romania still has one of the lowest employment rates for people with disabilities in Europe [15]; even if legislative frameworks (like Law No. 448/2006) ensure employment rights and reasonable accommodations, practical implementation faces significant challenges. Barriers include limited training opportunities, low employer awareness, pervasive stigma, and a lack of accessible infrastructure. 

The primary goal of the International Spinal Cord Injury (InSCI) Community Survey is to identify factors that explain the functioning and well-being of individuals with SCI across countries [16,17]. A total of 22 countries representing all six World Health Organization regions participated in this project [18]. This initiative, part of the Learning Health System for Spinal Cord Injury (LHS-SCI), is embedded in the WHO’s Global Disability Plan, aiming to collect statistical data on the lived experiences of people with SCI to inform recommendations and policy development [16,17]. The Romanian National Spinal Cord Injury Survey (RO-InSCI) represents the local adaptation of this community survey, designed to gather detailed information on the lived experiences of people with SCI in Romania.

Although Romania has allocated substantial EU and national funding to support employment for vulnerable groups, the impact has been limited. Public employment services struggle to provide effective job placement or follow-up support, and many individuals with disabilities are confined to sheltered employment rather than inclusive workplaces, contrary to the UN Convention on the Rights of Persons with Disabilities (CRPD).

To address these systemic issues, Romania must strengthen vocational training, raise employer awareness, enhance support mechanisms, and promote inclusive employment practices. Without these reforms, people with disabilities will remain marginalized in the labor market, perpetuating social and economic disparities [19].

Romania’s specific socioeconomic factors contribute to increased difficulties for SCI individuals in accessing rehabilitation, mobility aids, and specialized care. By focusing on Romania, this study aims to highlight the need for enhanced disability support systems and advocate for policy changes that would benefit individuals with SCI in Romania and similar regions with comparable challenges. The objectives of this study are to evaluate employment rates among Romanian participants with traumatic and non-traumatic SCI, and estimate the employment gap, which refers to the disparity or difference in employment rates between individuals with disabilities, such as those with spinal cord injuries (SCIs), and the general population. This will help to identify the main barriers to employment for SCI individuals, using surveys and the data available from the International Labour Organization Department of Statistics, and to examine differences in employment rates between those with traumatic SCI (TSCI) and non-traumatic SCI (non-TSCI).

## 2. Materials and Methods

The InSCI survey is a cross-sectional community-based study conducted across 22 countries from January 2017 to May 2019. Developed by Swiss Paraplegic Research in collaboration with representatives from participating InSCI countries, the survey used a standardized questionnaire, and each country followed standard operating procedures for data collection and management. National study centers were responsible for administering the survey within their respective countries, ensuring that the study protocol adhered to national laws and regulations and obtained Ethics Board approval as required. The study was conducted in compliance with the Declaration of Helsinki. Based on a power analysis, the InSCI study established a minimum sample size of 200 participants per country [16].

Inclusion criteria for the survey required participants to be adults (over 18 years) with either traumatic or non-traumatic SCI, living in the community, and able to understand and respond to the Romanian version of the questionnaire. Participants needed to provide informed consent voluntarily. Exclusion criteria included serious cognitive impairments or severe mental health conditions that could interfere with survey responses.

In Romania, the survey was coordinated by the National Study Center at the Rehabilitation Hospital Felix Spa, adhering to national legal standards, patient rights, and professional ethical norms. Ethical approval was granted by the Ethics Council of the Băile-Felix Medical Rehabilitation Clinical Hospital, and the study protocol was endorsed by the InSCI initiative (approval number: 2228/06.09.2017).

The recruitment process followed a convenience sampling strategy. Patients were invited to participate in the study by clinicians or rehabilitation staff familiar with the study protocol. Announcements were also made at patient organization events, such as workshops or meetings organized by the NGO Motivation Romania, which supports wheelchair users. Additionally, participants were directly invited based on databases from three public rehabilitation hospitals: Felix Spa Medical Rehabilitation Hospital, Emergency University Hospital Elias Bucharest, and Emergency County Hospital Sibiu.

For the current analysis, only Ro-InSCI participants of employable age, defined by the Organisation for Economic Co-operation and Development (OECD) as 18 to 65 years, were considered. Eligible participants were identified through multiple sources, including databases from three public rehabilitation hospitals in Romania (Felix Spa Medical Rehabilitation Hospital, Emergency University Hospital Elias Bucharest, and Emergency County Hospital Sibiu), as well as from the non-governmental organization Motivation Romania, which supports wheelchair users, including those with SCI. Recruitment was conducted using a targeted sampling strategy, with individuals invited to participate during visits to inpatient or outpatient clinics or during patient organization events. Those interested in the study completed the questionnaire on-site after providing informed consent.

Participants were asked to indicate whether movement or feelings were absent or abnormal only in the lower limbs (paraplegia) or in both the upper and lower limbs (tetraplegia), and whether they had complete or incomplete loss of movement and sensation below the level of SCI. From this, a variable type of injury was created with 2 groups: tetraplegics and paraplegics and complete vs. incomplete lesions, respectively. According to the cause of injury, we identified 2 groups of traumatic and non-traumatic etiology.

Employment status was assessed by asking participants to indicate one of the following options (multiple answers possible): (1) work for an employer, (2) work for an employer but currently on sick leave, (3) self-employed, (4) unpaid work in family business, (5) housekeeping, (6) student, (7) unemployed, (8) retired because of health problems, (9) retired because of age, or (10) other. Age, age at onset of SCI, and time since onset of SCI (TSI) were calculated from the years of birth, years since the onset of SCI, and year of completing the questionnaire. Response options for sex were male and female. Years of education were measured in line with the International Standard Classification of Education as the total years of formal education before and after onset of SCI, including school and vocational training [17].

Gaps in employment rates between people with SCI and the national general population were calculated as the difference between the observed employment rate for the study population and the general population among people of employable age in Romania, according to the International Labour Organization Department of Statistics (2017–2018) [18]. The differences in observed employment rates and employment gaps between traumatic and non-traumatic SCI were analyzed in the same way.

### Statistical Analysis

Socio-demographic data, SCI characteristics, and employment-related variables were analyzed and presented as percentages for categorical variables and means with standard deviations (SDs) for continuous variables, as appropriate. IBM SPSS Statistics software version 22.0 (IBM Corp., Armonk, NY, USA) was used to perform all statistical analyses.

Employment data were analyzed in terms of frequency and proportion. The chi-square test (χ^2^) was employed to assess associations between categorical variables, such as SCI etiology (traumatic vs. non-traumatic), injury level (tetraplegia vs. paraplegia), and employment status. The chi-square test is a non-parametric statistical test used to determine whether there is a significant association between two categorical variables. For instance, it helps identify whether employment status differs significantly across SCI etiologies or injury levels. A significant χ^2^ result indicates that the observed differences in frequencies are unlikely to be due to chance, providing evidence for potential relationships between the variables. This contextualizes the importance of the χ^2^ values reported, making the interpretation more meaningful.

For continuous variables, independent-sample *t*-tests were conducted to compare means between groups where applicable (e.g., average hours worked per week by injury level). The *t*-test determines whether there are statistically significant differences between the means of two groups. For example, it identifies whether individuals with paraplegia work significantly more hours on average than those with tetraplegia, highlighting variations in employment engagement based on injury level.

To calculate the employment gap, we determined the difference between the observed employment rate in the SCI population and the national employment rate for people of employable age in Romania, based on data from the International Labour Organization (ILO) for 2017–2018. This approach was also used to examine employment gaps between subgroups within the SCI population (traumatic vs. non-traumatic SCI).

A significance level of 0.05 was used for all statistical tests, and results with *p*-values below this threshold were considered statistically significant.

## 3. Results

This study includes 215 individuals with spinal cord injuries (SCIs), primarily male (72.09%), with a median age of 37 years (IQR: 30–46 years) at the time of survey participation. Most participants have completed secondary or vocational schooling, with a median of 12 years of education (IQR: 10–14 years). The main demographics that describe the Romanian survey participants are presented in Table 1.

### 3.1. Types of SCI and Etiological Factors

The median age at injury was 28 years (IQR: 21–38 years), indicating that the majority of injuries occurred in early adulthood—a factor that can significantly impact life trajectory and long-term outcomes. Paraplegia is more prevalent than tetraplegia, affecting 69.3% of participants compared to 30.7% with tetraplegia. Most participants (67.4%) have incomplete injuries, with 32.6% reporting complete injuries. In terms of etiology, traumatic causes account for 83.7% of SCIs, while non-traumatic causes represent 16.3%.

This distribution suggests that prevention efforts focused on trauma-related incidents, such as road accidents and workplace safety measures, could be effective in reducing SCI cases.

### 3.2. Demographic and Socioeconomic Characteristics Association with Injury Etiology

Gender distribution differs notably between traumatic and non-traumatic SCI. Traumatic SCI is more common among males (78.3%), whereas non-traumatic SCI is more prevalent among females (60%). Educational levels indicate that vocational school education is the most common in both groups, yet a higher percentage of non-traumatic SCI participants hold a bachelor’s degree (28.6%) compared to those with traumatic SCI (23.3%). This difference might reflect the different career paths associated with each type of injury. Regarding household income, non-traumatic SCI participants report higher income levels, with 34.3% having above-average income compared to 21.5% in the traumatic group, possibly indicating that individuals with non-traumatic SCI were employed in higher-paying jobs prior to their injury or managed to retain employment post-injury.

### 3.3. Employment Status Before and After SCI

**Figure 1:**
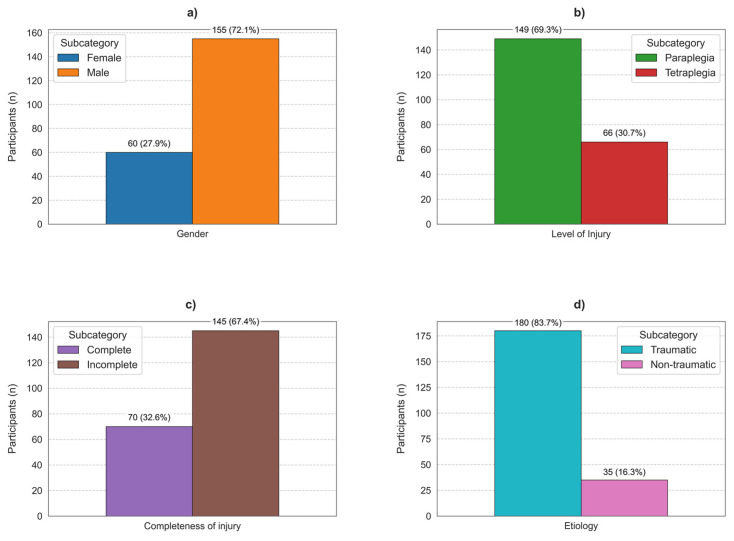
Demographic and injury characteristics of study participants with spinal cord injuries. (**a**) Gender distribution, (**b**) neurological level of injury, (**c**) completeness of injury, (**d**) etiology of injury.

Most participants (70.8%) were employed before their injury, with traumatic SCI participants slightly more likely to have been employed (70.1%) than those with non-traumatic SCI (60%). Among injury levels, paraplegic individuals were more likely to have held a job before their injury (69.3%) than quadriplegic individuals (67.7%). In contrast, 27.8% of the sample had no employment before injury, with a higher rate observed among non-traumatic SCI individuals (40%) and those with tetraplegia (32.3%). The main demographics are presented in Figure 1 and Table 2.

Following SCI, only 24.9% of participants were able to return to work, highlighting the significant employment challenges faced by SCI individuals. The observed employment rate of 25.35% contrasts with the national employment rate in Romania of 64.8%, resulting in an employment gap of 39.45%. The employment gap between individuals with SCI and the general population is significant, reflecting broader global trends.

Similar gaps have been observed in other countries, where people with disabilities face substantial barriers to employment, including discrimination, lack of accessible work environments, and insufficient vocational rehabilitation support. For example, in the United States, the employment rate for individuals with disabilities is approximately 43.3% lower than for those without disabilities, as reported by the U.S. Bureau of Labor Statistics in 2023 [20]. Similarly, in Germany, the employment rate for people with disabilities is significantly lower than the general population, with a reported difference of 41.2% in 2019, despite efforts to improve workplace accessibility and inclusivity [21]. In some countries, like Morocco and South Africa, the overall low employment rates in the general population contributed to relatively smaller employment gaps, despite the low employment rates among individuals with SCI. Additionally, while some countries showed observed employment rates that closely aligned with predicted rates, substantial deviations were noted in others. The most pronounced negative differences were recorded in Brazil, Greece, Morocco, Romania, and Spain [13]. These global trends highlight the need for systemic changes, such as improved accessibility, employer incentives, and enhanced vocational training, to reduce employment disparities and improve social inclusion for individuals with SCI worldwide.

After exploring the demographic characteristics of individuals with spinal cord injuries (SCIs), it is essential to examine how these factors intersect with employment outcomes. Understanding the demographic profile provides crucial insights into the barriers and opportunities that influence employment status for SCI individuals. Before injury, the most common job category was elementary occupations (50% in traumatic SCI and 31.6% in non-traumatic SCI), with paraplegic individuals more frequently holding these roles. Office support work was the second most common category, especially among non-traumatic SCI participants (31.6%) and quadriplegic individuals (26.2%). A small number of participants held professional or managerial positions prior to injury, with a slightly higher percentage among paraplegic individuals (15.2%) compared to quadriplegic individuals (4.8%).

### 3.4. Vocational Rehabilitation Participation

The majority of participants (79.9%) did not receive vocational rehabilitation after their SCI. This rate was slightly higher among traumatic SCI participants (82.5%) compared to non-traumatic SCI participants (77.1%). Only 18.7% of participants benefited from vocational rehabilitation services, indicating limited accessibility or availability of such support for SCI individuals in Romania (Table 3).

The low participation rate in vocational rehabilitation among individuals with SCI is concerning and may be influenced by factors such as limited awareness of available programs, inefficacy of services, and physical or psychological barriers. To improve these rates, targeted programs could help increase awareness, while adapting the services to the specific needs of SCI individuals—such as offering remote or flexible options—may enhance engagement. Additionally, integrating vocational rehabilitation services with healthcare providers could facilitate early referral and increase participation [9,22,23].

### 3.5. Desire to Return to Work

When asked about their desire to return to work post-SCI, 82% of traumatic SCI participants expressed an interest, compared to 61.1% in the non-traumatic group (χ^2^ = 7.574, *p* < 0.005\chi^2^ = 7.574, *p* < 0.005, χ^2^ = 7.574, *p* < 0.005), suggesting that individuals with traumatic SCIs may have higher aspirations for reintegration into the workforce (Table 4). Among injury levels, 76.9% of tetraplegic participants and 79.1% of paraplegic participants expressed a desire to work. This small difference was not statistically significant, indicating a shared interest across injury levels in resuming employment if feasible.

### 3.6. Barriers to Employment by Etiology and Injury Level

Barriers to employment were reported in relation to both SCI etiology and injury level. Inadequate transport and access to infrastructure significantly limit daily life and employment for individuals with SCI. Without accessible transport, daily life activities become challenging, reducing job retention and social participation. Similarly, inaccessible buildings and public spaces decrease employment opportunities, reinforcing social isolation and economic discrepancies. Health status and disability were cited by both traumatic (52.8%) and non-traumatic SCI participants (57.1%), with no significant difference between the two groups (χ^2^ = 4.3, *p* = 0.64\chi^2^ = 4.3, *p* = 0.64, χ^2^ = 4.3, *p* = 0.64). Inadequate transport was a significant barrier for traumatic SCI individuals, with 25% reporting it as an issue compared to only 2.9% of non-traumatic SCI participants (*p* = 0.003), indicating a significant accessibility challenge for this group. Insufficient access to infrastructure also emerged as a barrier more frequently among traumatic SCI participants (23.9%) compared to non-traumatic (8.6%), with a statistically significant difference (*p* = 0.04, *p* = 0.04, *p* = 0.04). A higher percentage of non-traumatic SCI participants (5.7%) cited “other reasons” for not working, which was significantly different from the traumatic group (0.6%; *p* = 0.02, *p* = 0.02, *p* = 0.02).

Comparing barriers by injury level, health or disability-related challenges were more common among tetraplegic participants (62.1%) than paraplegic participants (49.7%), although this difference was not statistically significant (χ^2^ = 12.4, *p* = 0.09\chi^2^ = 12.4, *p* = 0.09, χ^2^ = 12.4, *p* = 0.09). More paraplegic participants (23.5%) reported access to infrastructure as a barrier compared to tetraplegic participants (16.7%), although this difference was also not statistically significant. Concerns regarding the potential loss of financial benefits were similar for both groups, with no significant difference (χ^2^ = 1.5, *p* = 0.96\chi^2^ = 1.5, *p* = 0.96, χ^2^ = 1.5, *p* = 0.96). Only a small proportion of participants expressed a lack of desire to work, with low rates for both tetraplegia (3%) and paraplegia (4.7%), suggesting a general willingness to work if barriers were addressed (Table 5).

The findings underscore the significant barriers to employment faced by SCI individuals, including health-related challenges, inadequate transport, and access to infrastructure issues, particularly among those with traumatic SCI. The limited participation in vocational rehabilitation highlights the need for improved accessibility and awareness of such programs. Additionally, the considerable employment gap between SCI individuals and the general population in Romania underscores the importance of inclusive labor market policies to support workforce reintegration and address the specific needs of SCI individuals.

## 4. Discussion

### 4.1. Barriers to Employment

Employment is critical for the quality of life and social inclusion of individuals with SCI. Yet, numerous studies reveal that achieving meaningful employment post-SCI requires access to a supportive work environment and effective vocational rehabilitation programs, resources that are often limited or inaccessible [24]. Employed status significantly contributes to financial independence, social inclusion, and overall well-being. Access to meaningful employment not only improves economic stability but also enhances mental and emotional health by creating a sense of purpose and belonging. Studies have shown that employment can significantly reduce the risk of depression and anxiety among SCI individuals by providing a sense of purpose and control over their lives. A very recent study published in Nature by Escorpizo et al. (2024) found that SCI patients who were employed reported higher levels of life satisfaction and social engagement compared to their unemployed counterparts [25]. Furthermore, SCI patients are more likely to form positive relationships and integrate into their communities when they participate in the workforce [26]. These findings highlight the vital role of employment in enhancing both the psychological and social well-being of SCI individuals.

Recognized globally as a fundamental right, the right to work for persons with disabilities is protected under the United Nations Convention on the Rights of Persons with Disabilities. This convention strives to establish a universal legal framework that guarantees equality, non-discrimination, and access to professional opportunities for persons with disabilities, affirming their right to freely choose their employment paths [27].

Romania’s legislative measures have sought to align with this global vision, aiming to support the integration and social inclusion of people with disabilities by ensuring their right to equal employment access. Romanian Law 448/2006 (updated in emergency ordinance 47/2023) seems to provide a robust framework to promote the employment and inclusion of individuals with disabilities. The law ensures the right of individuals with disabilities to work in roles aligned with their abilities, requiring employers to uphold equality in recruitment, training, and promotion processes. It also establishes an employment quota system, obligating companies with 50 or more employees to allocate at least 4% of their workforce to individuals with disabilities. To encourage hiring, the law offers incentives such as tax exemptions, subsidies for adapting workplaces, and financial support for vocational training. Employers are also obligated to adapt workplaces to ensure accessibility and eliminate barriers, with strict prohibitions against discrimination in employment practices. Further support is provided for self-employment and entrepreneurship through financial aid and professional guidance, alongside programs for vocational rehabilitation to enhance employability [24].

These measures underline Romania’s commitment to creating an inclusive and supportive labor market for individuals with disabilities. However, despite this legal backing, the actual employment experience of individuals with SCI in Romania highlights several gaps. The journey toward social and economic inclusion for individuals with SCI is complex and often fraught with barriers, particularly in the realm of employment.

### 4.2. Role of Vocational Rehabilitation

This discussion addresses the economic and social realities faced by individuals with SCIs, focusing on the obstacles to employment and the effectiveness of vocational support systems. The insights gathered from the International Spinal Cord Injury Survey (InSCI) underscore the importance of understanding the biopsychosocial barriers to employment for SCI individuals, as emphasized by the WHO’s International Classification of Functioning, Disability, and Health (ICF) model. This model frames SCI employment challenges within a broader context of health, social, and environmental factors, offering a multidimensional perspective on the reintegration of SCI individuals into the workforce [11].

Building on the biopsychosocial framework, employment, along with participation in income-generating activities, is a cornerstone of social inclusion and has been consistently linked to enhanced quality of life for individuals with SCIs [5]. However, SCI participants in fields such as elementary labor, agriculture, and military roles are particularly vulnerable, often due to the physically demanding nature of these jobs and the lack of protective measures [28]. The situation is further complicated by a reported lack of vocational rehabilitation support, with limited access to services such as counseling, retraining, and qualification courses. Although some individuals in Romania have accessed these services, their reach and availability remain inadequate. One solution to address these shortages is to incorporate successful policy models from other countries for improving vocational rehabilitation and workplace integration in Romania. For example, the Swedish vocational rehabilitation system has been highly effective, focusing on early intervention, personalized training, and employer partnerships to support individuals with disabilities in returning to work [29,30]. Also, Canada’s Workplace Integration Program provides financial help for employers to adapt their workplaces, alongside comprehensive training programs, resulting in higher employment retention rates for disabled individuals [31,32]. By learning from these international models, Romania could strengthen its vocational rehabilitation efforts, ensuring better integration and long-term employment success for people with disabilities.

Comparing Romania’s findings to those from other countries included in the InSCI survey offers a broader perspective on the challenges faced by individuals with SCIs. While Romania shares many common barriers with other countries, such as limited access to vocational rehabilitation and inadequate workplace accommodations, some unique challenges emerge. For instance, Romania’s relatively lower rates of employment and vocational rehabilitation participation among SCI individuals could reflect differences in the availability of financial support and infrastructure, compared to countries like Germany or Sweden, where more robust vocational training programs and employer incentives are in place. Conversely, Romania’s efforts to improve disability benefits and social support align with global trends, where comprehensive social security systems help mitigate some of the financial burdens of SCI. By highlighting these similarities and differences, we can better understand the effectiveness of Romania’s policies and identify areas for improvement by learning from successful practices worldwide.

### 4.3. Economic Implications

Employment rates post-SCI remain notably low, reflecting the complex interplay of injury-related factors, environmental limitations, and systemic issues. While many individuals with SCI express a strong desire to return to work, especially those with traumatic injuries and quadriplegia, the reality of doing so is often hampered by insufficient infrastructure, lack of accessible transportation, and limited workplace accommodations. Additionally, systemic barriers such as rigid work schedules, inadequate assistive technologies, health challenges, and insufficient rehabilitation services create significant obstacles to successful workforce reintegration.

The observed differences in socioeconomic outcomes between traumatic and non-traumatic spinal cord injury (SCI) groups can be explained by several factors. Non-traumatic SCIs often occur in individuals with pre-existing medical conditions, who may already have established careers or more stable financial situations prior to the onset of their injury. In contrast, traumatic SCIs are more frequently associated with younger individuals, who may still be in the early stages of their careers, thus impacting their long-term earning potential [9,33,34].

Additionally, the lower rates of vocational rehabilitation among traumatic SCI individuals may be related to the sudden nature of their injuries, which can have a profound psychological impact. This may lead to longer periods of adjustment and reduced readiness to return to work compared to non-traumatic cases, which may develop more gradually. Furthermore, access to vocational services may differ based on the cause of injury, as non-traumatic SCIs might be more likely to occur in individuals covered by more robust health insurance, whereas traumatic SCIs may more frequently rely on limited insurance options [9,33,34].

For individuals with SCIs in Romania, financial support alone does not suffice to meet the elevated cost of living associated with this condition. The expenses of medical care and rehabilitation often exceed those faced by the general population, exacerbated by gaps in healthcare and social assistance policies [35]. On average, medical care for SCI patients can range from hundreds to thousands of RON per day, depending on the severity of the injury and the need for long-term treatments. Rehabilitation programs, including physical therapy and vocational retraining, also add to the financial burden. In contrast, the average net income in Romania is approximately RON 5300 per month, while social support for disabled individuals, such as disability pensions or allowances, may be insufficient to cover these costs. This economic strain contributes to a precarious financial situation for many SCI individuals and their families, fueling the risk of social exclusion—a phenomenon that correlates with higher mortality rates among those with SCIs [36]. Nevertheless, evidence from international studies consistently shows that individuals with SCIs can perform a wide range of jobs effectively when given appropriate training, rehabilitation, and workplace support [6].

### 4.4. Limitations and Further Perspectives

As part of the first global SCI survey, the RO-InSCI study presents the first employment rate data for Romania.

However, the study has several limitations. It relied on a convenience sampling frame, consistent with the methodology used by most countries participating in the InSCI survey. Moreover, the absence of information on the basic characteristics of non-respondents precluded the evaluation of response bias. As a result, the reported employment rates should not be considered unbiased estimates of employment among individuals with SCI. Additionally, all data were self-reported, leading to some missing values and the potential for inaccuracies or recall bias, particularly regarding the reporting of SCI characteristics.

Another limitation of this study is the relatively small sample size of non-traumatic SCI individuals (N = 35), which may impact the generalizability of the findings. While the data collected offer valuable insights into this subgroup, the limited number of participants reduces the ability to give general conclusions about the experiences of non-traumatic SCI individuals in the wider population. However, it is important to recognize that non-traumatic SCIs are inherently less prevalent compared to traumatic SCIs, making it challenging to achieve larger sample sizes in studies focusing on this population. Future research with larger, more diverse samples is necessary to validate these findings and explore additional factors that may influence outcomes for non-traumatic SCI individuals.

A notable strength of the study is its calculation of employment gaps by comparing the results to those of the general national population. This discussion will delve further into the specific challenges faced by individuals with SCIs in Romania, analyzing the barriers to employment, the role of vocational rehabilitation, and the broader socioeconomic implications of SCIs. It aims to highlight actionable strategies and policy recommendations to enhance the inclusivity and accessibility of the Romanian labor market for persons with SCIs.

Future research should explore several key areas to further understand and improve outcomes for SCI patients. Longitudinal studies on the effectiveness of vocational rehabilitation programs could provide valuable insights into their long-term impact on employment, mental health, and overall life satisfaction. Additionally, investigating employer perspectives on hiring persons with SCIs could help discover ways to improve workplace integration. These studies could contribute to increasing social inclusion and economic independence.

## 5. Conclusions

This study highlights the significant employment challenges faced by individuals with SCIs in Romania, underscoring the need for targeted interventions to improve their inclusion in the workforce.

Employment rates among individuals with SCIs are significantly lower than those of the general population. The majority of SCI individuals in the community remain unemployed, with those lacking formal education or previously engaged in physically demanding jobs, such as elementary occupations, being particularly affected. The discrepancies between observed and predicted employment rates indicate that healthcare, rehabilitation, and employment systems, along with relevant policies, play a crucial role in shaping employment outcomes.

Enhanced vocational rehabilitation services, flexible work arrangements, and accessible transportation and infrastructure are crucial to enable SCI individuals to participate fully in the labor market. Increasing the quality and accessibility of medical rehabilitation services is also critical to ensure the long-term health and functional capacity of individuals with SCIs. Adopting the guidelines of the United Nations Convention on the Rights of Persons with Disabilities at the national level, to ensure unrestricted work access for people with disabilities, is essential not only for economic reasons but also to counteract social exclusion.

Our findings suggest that tailored employment policies are essential to address the physical and social barriers experienced by people with SCIs, promote professional retraining, and enable interdisciplinary rehabilitation strategies to support sustainable workforce reintegration.

Addressing these needs through comprehensive, inclusive labor policies can foster better employment outcomes, reduce social isolation, and ultimately improve the quality of life for individuals with SCIs in Romania. Also, addressing these barriers is essential not only for economic and financial reasons, but for creating real opportunities for social inclusion and equity for individuals with SCIs.

## Figures and Tables

**Table 1:** Demographic and lesion characteristics of participants in Ro-InSCI survey.

Total Number	Gender		Median Age (Years) at Time of the Survey		Median Years of Education	Age at Injury (Years)		Level of Injury	Completeness of Injury		Etiology
215	Female (%) 60 (27.91)	Male (%) 155 (72.09)	37 (30, 46)	12 (10, 14)	28 (21, 38)	Paraplegia 149 (69.3)	Tetraplegia 66 (30.7)	Complete 70 (32.6)	Incomplete 145 (67.4)	Traumatic 180 (83.7)	Non-traumatic 35 (16.3)

**Table 2:** Demographic and socioeconomic characteristics of individuals with traumatic and non-traumatic spinal cord injuries.

Variables		Traumatic Etiology N = 180	Non-Traumatic Etiology (N = 35)
Gender	Female	39 (21.7%)	21 (60%)
	Male	141 (78.3%)	14 (40%)
Marital status	Unmarried	96 (53.3%)	11 (31.4%)
	Married	71 (39.4%)	18 (51.4%)
	Divorced/widowed	13 (7.2%)	6 (17.1%)
Educational level	Gymnasium	47 (27.3%)	9 (25.7%)
	Vocational school	63 (36.6%)	13 (37.1%)
	High school	22 (12.8%)	3 (8.6%)
	Bachelor	40 (23.3%)	10 (28.6%)
Household income	Very low income	47 (26.6%)	9 (25.7%)
	Low income	46 (26%)	6 (17.1%)
	Average income	46 (26%)	8 (22.9%)
	Above average income	38 (21.5%)	12 (34.3%)

**Table 3:** Employment and vocational rehabilitation status of individuals with spinal cord injuries by etiology and level of injury.

Variables	Total	SCI Etiology		Level of Injury	
		Traumatic (N = 180)	Non-Traumatic (N = 35)	Quadriplegic (N = 66)	Paraplegic (N = 149)
	Work before SCI				
No job before SCI	58 (27.8%)	50 28.2%	14 40%	21 32.3%	43 29.3%
Job before SCI	148 (70.8%)				
No answer	3	3 1.7%	0	1 1.5%	2 1.3%
	ISCO job title before SCI				
Managers		11 9%	1 5.3%	3 7.1%	9 9.1%
Professionals		13 10.7%	4 21.1%	2 4.8%	15 15.2%
Technicians and Associate Professionals		14 11.5%	1 5.3%	4 9.5%	11 11.1%
Office support workers		19 15.6%	6 31.6%	11 26.2%	14 14.1%
Service and sales workers		0	0	0	0
Skilled workers in agriculture, forestry and fishing		3 2.5%	0	1 2.4%	2 2%
Crafts from related trades		1 0.8%	1 5.3%	0	2 2%
Plant and machine operators and fitters		0	0	0	0
Elementary occupations		61 50%	6 31.6%	21 50%	46 46.5%
Armed forces		0	0	0	0
No answer		58 32.2%	16 45.7%	24 36.4%	50 33.6%
	Vocational rehabilitation				
No	167 (79.9%)	146 82.5%	27 77.1%	53 80.3%	120 82.2%
Yes	39 (18.7%)	31 17.5%	8 22.9%	13 19.7%	26 17.8%
No answer	3	3 1.7%	0	0	3 2%

**Table 4:** Comparisons according to etiology (traumatic versus non-traumatic) and level of injury (tetraplegia versus paraplegia) regarding the desire to be reinstated at work.

**Variables**	**SCI Etiology**		**df.**	**χ^2^**	***p***
	**Traumatic**	**Non-Traumatic**			
Desire for a job and salary	82%	61.1%	20.9	7.574	0.005
**Variable**	**Injury Level**		**df.**	**χ^2^**	***p***
	**Tetraplegia**	**Paraplegia**			
Desire for a job and salary	76.9%	79.1%	7.8	1.837	0.17

**Table 5:** Comparisons according to etiology (traumatic versus non-traumatic) and level of injury (tetraplegia versus paraplegia) regarding the reasons for not working.

**Items**	**SCI Etiology**		**df.**	**χ^2^**	***p***
	**Traumatic**	**Non-Traumatic**			
Health status/disability	52.8%	57.1%	4.3	0.217	0.64
Education/vocational training	6.7%	2.9%	3.8	0.737	0.39
Family responsibilities	3.9%	5.7%	1.8	0.235	0.62
No suitable job	27.8	22.9	4.9	0.355	0.55
Limited job search skills	11.7%	5.7%	6	1.097	0.29
No financial needs	2.2%	0%	0	0	0
Parents/spouse forbid work	0.6%	0%	0	0	0
Inadequate means of transport	25%	2.9%	21.1	8.468	*0.003*
Insufficient access to infrastructure	23.9%	8.6%	15.3	4.058	*0.04*
Absence of assistive devices	11.7%	2.9%	8.8	2.451	0.11
Fear of loss of financial benefits (disability allowance)	4.4%	11.4%	7	2.731	0.09
I don’t want to work	3.3%	8.6%	5.3	2.054	0.15
Other reasons	0.6%	5.7%	5.1	5.381	*0.02*
**Items**	**Injury Level**		**df.**	**χ^2^**	***p***
	**Tetraplegia**	**Paraplegia**			
Health status/disability	62.1%	49.7%	12.4	2.814	0.09
Education/vocational training	6.1%	6%	0.1	0.001	0.97
Family responsibilities	6.1%	3.4%	2.7	0.819	0.36
No suitable job	27.3%	26.8%	0.5	0.006	0.93
Limited job search skills	10.7%	10.6%	0.1	0.0001	0.98
No financial needs	3%	1.3%	1.7	0.736	0.39
Parents/spouse forbid work	0%	0.7%	0.7	0	0
Inadequate means of transport	22.7%	20.8%	1.9	0.098	0.75
Insufficient access to infrastructure	16.7%	23.5%	6.8	1.251	0.26
Absence of assistive devices	10.6%	10.1%	0.5	0.012	0.91
Fear loss of financial benefits	4.5%	6%	1.5	0.196	0.96
I don’t want to work	3%	4.7%	1.7	0.329	0.56
Other reasons	1.5%	1.3%	0.2	0.014	0.90

## Data Availability

The data supporting this study are available upon reasonable request from the corresponding author.
